# Malicious Legislation

**Published:** 1911-11-15

**Authors:** 


### MALICIOUS LEGISLATION.

IN THE SENATE OF THE UNITED STATES.

APRIL 20, 1911.

MR. CULLOM introduced the following bill; which was read twice and referred to the Committee on Patents.

A BILL

To renew and extend certain letters patent.

*Be it enacted by the Senate and House of Representa-*

*tives of the United States of America in Congress assembled,* That certain letters patent for an alleged new and useful improvement in dentistry, dated March fifteenth, eighteen hundred and eighty-one, and Numbered Two hundred and thirty-eight thousand nine hundred and forty, granted to James E. Low, be, and the same is hereby, renewed and extended to James E. Low for the term of seventeen years from and after the passage of this Act.

Hon. James R. Mann,
Washington, D. C.

Dear Congressman Mann:

Enclosed herewith please find Senate Bill No. 1342, introduced by Senator S. M. Cullom of Illinois, extending certain letters patent for crowns and bridges to James E. Low for a period of seventeen years after the passage of this act.

Please note this patent expired March 15th, 1899 (twelve years ago.) While on the surface this bill seems harmless, it is a vicious and far reaching measure, being promoted by a few unscrupulous persons, who wish to extort royalties from the 40,000 dentists in the United States, and is very unjust to every person requiring dental service.

Before any such bill is passed, I urge your most careful consideration and full investigation of the subject. We also hope for a full hearing on the matter. The above is the sentiment of every dentist in the country.

Sincerely yours,

J. W. Shedd.

Chicago, September 11, 1911.

Dr. J. W. Shedd,
132 North Wabash Ave., Chicago.

My dear Dr. Shedd:

I have your kind favor in reference to the Cullom Bill, Senate Bill No. 1342, introduced by Senator Cullom at the recent session of Congress, to extend the James E. Low patent.

I am very much surprised that Senator Cullom should introduce this bill. I do not believe it has any merit. I looked into the matter once and concluded it would be a gross abuse of power for Congress to pass the bill. A similar bill has been urged for several years. I shall certainly fight the bill and I feel sure it will not be passed by Congress. While ordinarily I do not like to express opinions in advance, yet this bill is so flagrantly bad that I promise you it will meet with determined opposition if it comes up in the House of Representatives while I am a member. I again express surprise that Senator Cullom should have introduced it.

Yours very sincerely,
JAMES R. MANN.

1350 First National Bank Bldg.

The above correspondence is of interest to every dentist and needs the concerted action of all to prevent its passage. Inform your congressman of its intent and the injustice of such a measure. It should be prevented by every proper influence that can be brought to bear upon our congressmen.

